# GeDi: applying suffix arrays to increase the repertoire of detectable SNVs in tumour genomes

**DOI:** 10.1186/s12859-020-3367-3

**Published:** 2020-02-05

**Authors:** Izaak Coleman, Giacomo Corleone, James Arram, Ho-Cheung Ng, Luca Magnani, Wayne Luk

**Affiliations:** 1 Department of Computing, Imperial College London, London, SW7 2AZ UK; 2 Systems Biology PhD Program, Columbia University in New York City, New York, USA; 3 Department of Surgery and Cancer, Imperial College Hammersmith, London W12, UK

**Keywords:** Variant calling, SNV, Cancer, Genomics, Suffix array

## Abstract

**Background:**

Current popular variant calling pipelines rely on the mapping coordinates of each input read to a reference genome in order to detect variants. Since reads deriving from variant loci that diverge in sequence substantially from the reference are often assigned incorrect mapping coordinates, variant calling pipelines that rely on mapping coordinates can exhibit reduced sensitivity.

**Results:**

In this work we present GeDi, a suffix array-based somatic single nucleotide variant (SNV) calling algorithm that does not rely on read mapping coordinates to detect SNVs and is therefore capable of reference-free and mapping-free SNV detection. GeDi executes with practical runtime and memory resource requirements, is capable of SNV detection at very low allele frequency (<1%), and detects SNVs with high sensitivity at complex variant loci, dramatically outperforming MuTect, a well-established pipeline.

**Conclusion:**

By designing novel suffix-array based SNV calling methods, we have developed a practical SNV calling software, GeDi, that can characterise SNVs at complex variant loci and at low allele frequency thus increasing the repertoire of detectable SNVs in tumour genomes. We expect GeDi to find use cases in targeted-deep sequencing analysis, and to serve as a replacement and improvement over previous suffix-array based SNV calling methods.

## Background

Raw NGS data consist of million of sequencing reads derived from unknown genomic location. To detect SNVs in paired tumour-control NGS datasets, SNV calling pipelines must compare reads of the tumour dataset against reads of the control dataset that derive from the same genomic location. Accordingly, this requires organising the input data by genomic location. Current popular somatic SNV calling pipelines organise the input data by mapping tumour and control reads to a human reference genome prior to SNV detection. Once mapped, a downstream somatic SNV caller utilises the mapping coordinates to examine reads covering the same location across the tumour and control datasets, and ultimately detect SNVs. We categorise SNV callers that rely on mapping coordinates - and therefore a reference genome - to detect SNVs as *reference-based SNV callers*. A considerable drawback of reference-based SNV callers is the reduced sensitivity they exhibit in the presence of incorrect mapping coordinates. This limits their ability to characterise complex variant loci (variant loci other than sparsely distributed SNVs) where incorrect mapping coordinates arise frequently [1, 2].

The aforementioned drawback of reference-based SNV callers motivated development of SMuFin [3], a somatic SNV caller that utilises a generalised suffix array data-structure (*suffix array*) to detect SNV both mapping-free and reference-free; we call this approach *suffix array-based SNV detection*, and we therefore classify SMuFin as a *suffix array-based SNV caller*. Since suffix array-based SNV detection is reference-free and mapping-free, the approach is capable of detecting SNVs at complex variant loci. Consequently, it has potential to increase the repertoire of detectable SNVs in tumour genomes by enabling characterisation of such loci.

Although suffix array-based SNV detection has attractive features, its implementation in SMuFin suffers a number of shortcomings: Firstly, SMuFin’s available source code is inoperative to any useful degree - despite considerable effort from our group, SMuFin crashed without output when analysing all but one dataset; a small-scale simulated dataset available on SMuFin’s web-page. Secondly, SMuFin shows poor runtime and memory requirements, one group reporting a runtime of over 30 days to analyse a 30x whole genome dataset [4]. Finally, SMuFin’s recall for SNV detection is low, ranking in 17th place among 18 popular SNV calling pipelines when analysing a 30x Whole Genome Sequencing dataset [5].

The work herein provides three major contributions:

- Firstly, we introduce **Ge**neralised Suffix Array-based **Di**rect SNV caller, or **GeDi**, pronounced ‘Jeh-dye’ (“Implementation” section): A C++ implementation of a suffix array-based SNV caller. Like SMuFin, GeDi does not rely on mapping coordinates to detect SNVs, and can therefore detect SNVs both mapping- and reference-free. Accordingly, our work re-introduces an operative suffix-array based SNV caller for use somatic variant calling pipelines.
- Secondly, we design a novel approach to suffix array-based SNV detection and implement this in GeDi. Our approach makes use of an optional preprocessing filter to dramatically reduce GeDi’s runtime and memory usage (“Preprocessing” section), and a dual suffix array design that enables GeDi to detect *rare SNVs* (SNVs occurring at an allele frequency of ≤ 5%) with high precision (“Suffix array-based SNV detection” section).
- Finally, we extensively evaluate GeDi’s recall, precision and resource requirements when analysing real and simulated datasets (“Results” section). Our findings show: GeDi’s runtime and memory resource requirements are very practical, being lower than those of SMuFin and MuTect - a popular reference-based SNV caller [6] - in almost all test cases; GeDi is capable of calling rare SNVs and SNVs belonging to clustered hypermutations (loci with densely packed SNVs) with high precision, in contrast, MuTect grossly under-characterises these events; when analysing a previously published WGS dataset, GeDi detected a large number of previously unpublished SNVs located in functional genomic regions, many of which reveal putative sites of clustered hypermutation.

The following sub-section describes suffix-array based SNV detection as implemented in SMuFin, which forms the basis of GeDi’s novel approach.

### Previous approach

We distinguish between *SNV detection*, where SNVs and reads covering them are identified within tumour-control NGS datasets, and *SNV calling*, where detected SNVs’ structure and reference genome coordinates are computed and reported back to the user.

Unlike reference-based SNV callers that rely on mapping to organise input reads by genomic location, SMuFin uses a (generalised) suffix array constructed from the input reads [3]. This suffix array, being a lexicographically sorted array of the input read’s suffixes, imparts the property that all suffixes sharing a common prefix are contiguous within the array. Once constructed, this property allows splitting of the suffix array into *sections*: intervals containing suffixes with a common prefix of length ≥ 30. Suffixes within a section are considered to derive from the same genomic location due to the presence of the common prefix [3]. Hence, constructing a suffix array and splitting it into sections organises the information contained within the input reads by genomic location without use of a reference or mapping; the organisation is achieved during suffix array construction where the data is compared directly to itself.

**Fig. 1:**
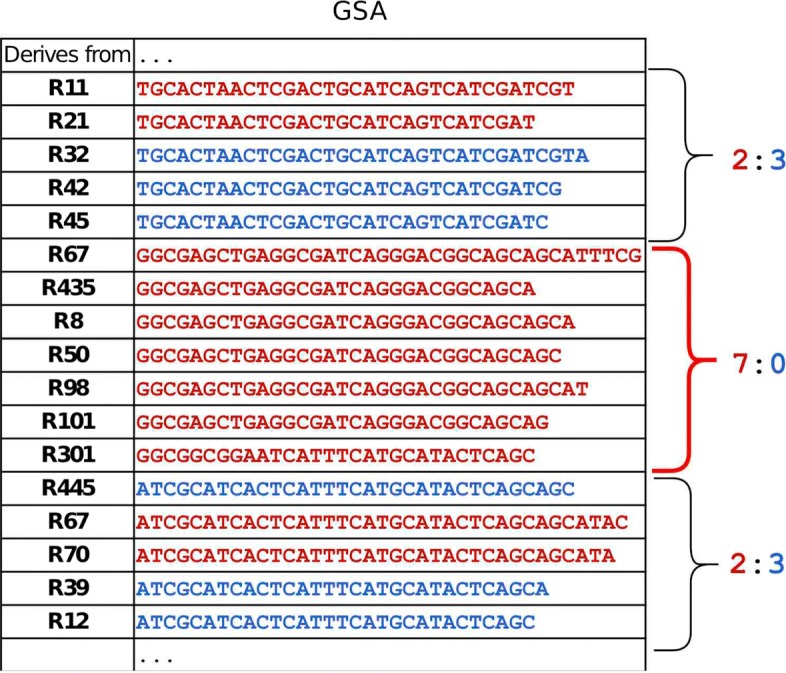
Left column shows the unique read identifier from which each suffix within the shown interval derives. Red and blue suffixes derive from the tumour and control datasets respectively. Three sections are shown, each bounded by a curly brace. Red and blue numbers next to each section represent the quantity of tumour- and control-read-derived suffixes within it respectively. The middle section (red curly brace) is enriched for tumour-read-derived suffixes and contains ≥*MSS* reads. Accordingly, tumour reads containing a suffix within this section are extracted for downstream SNV calling

Once the suffix array is split into sections, SNVs are detected by inspecting each section. SMuFin keeps track of each suffix’s derivation, either tumour- or control-read-derived, within the array, enabling the two types to be quantified within a section (Fig. 1). SNV detection is based on the inference that sections enriched for tumour-read-derived suffixes are likely to cover SNVs since, barring contamination, SNVs are exclusive to the tumour-read-derived NGS dataset. SMuFin considers a section enriched for tumour-read-derived suffixes if two conditions are satisfied: First, within the section, the number of tumour-read-derived suffixes divided by the total number of suffixes is greater than *e*_*cont*; a user-defined parameter that allows for the presence of tumour-cell contamination in control tissue. Second, the number of tumour suffixes within the section is at least Minimum Suffix Size (*MSS*=4); a constraint imposed to avoid mis-classification of sequencing errors (unique to the tumour-tissue derived dataset) as SNVs. Once enriched sections are identified (e.g middle section, Fig. 1), tumour reads containing suffixes within enriched sections are extracted. After this stage, SNVs and reads covering them have been identified within the tumour-control NGS datasets, thus completing SNV detection.

## Implementation

**Fig. 2:**
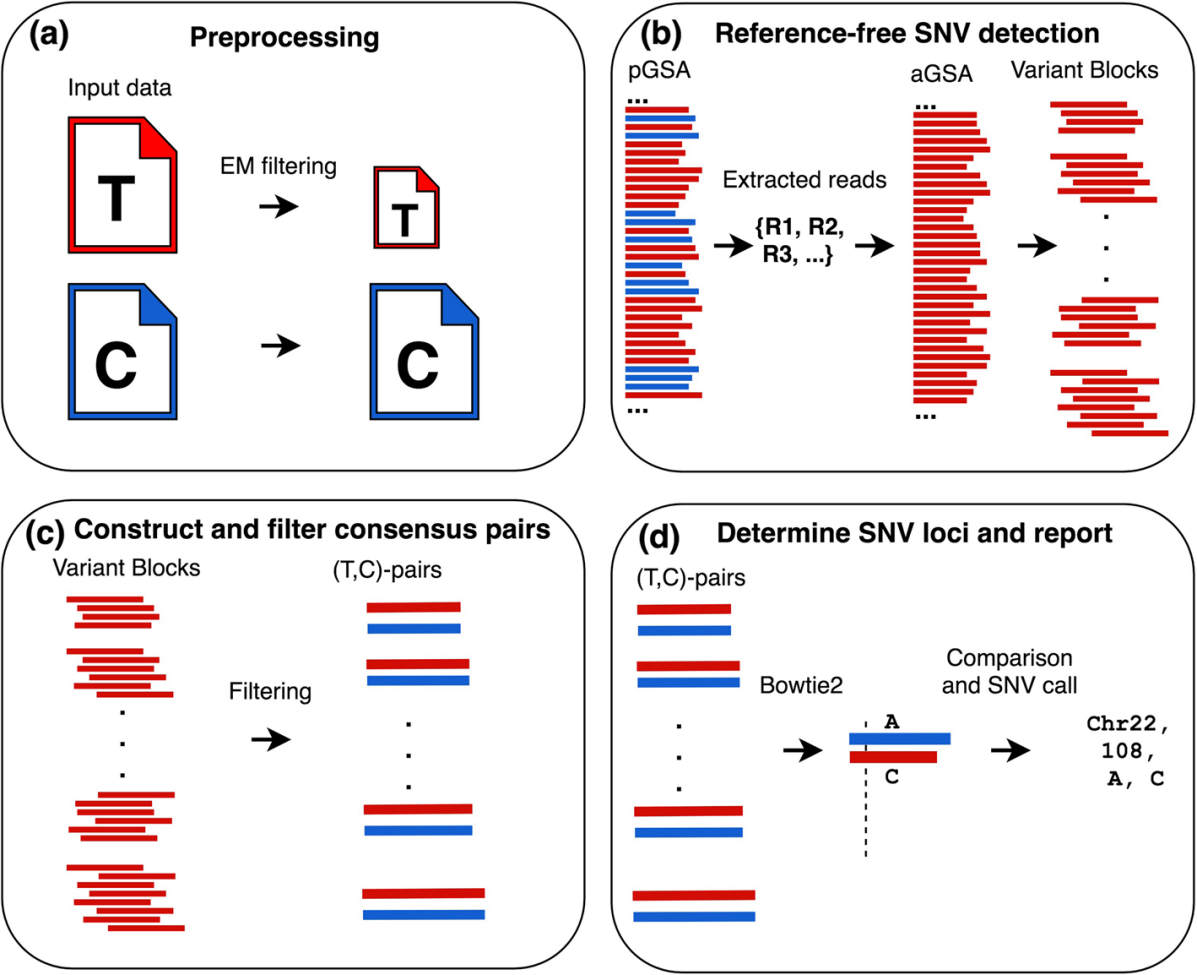
Overview of GeDi algorithm. pGSA and aGSA denote GeDi’s primary and auxiliary suffix arrays respectively. Data derived from tumour and control NGS datasets are given red and blue colouration respectively. **a** First, preprocessing filters out tumour reads that exactly match the reference and removes ‘N’ characters from the input tumour and control data (red T and blue C files respectively). **b** Second, suffix array-based SNV detection uses a dual suffix array design to detect SNVs, including those at low allele frequency. Variant blocks are constructed. **c** Third, consensus pairs (labelled T,C-pairs in diagram) are constructed from variant blocks and from control reads covering the same genomic location. False positives are removed by consensus pair filtering. **d** Finally, SNVs are called using control consensus pairs as proxies to compute SNV genome coordinates

In this section we introduce GeDi, describing its architecture as five stages of execution: Preprocessing, suffix array-based SNV detection, consensus pair construction, consensus pair filtering, and SNV calling. Figure 2 provides a simplified graphical overview of GeDi’s architecture.

### Preprocessing

In this stage, GeDi reduces the input data size and removes ‘N’ characters (Fig. 2a).

Suffix array construction is the most resource intensive computation of suffix array-based SNV detection. To combat this, we developed a preprocessing filter, *emfilter*, and apply emfilter immediately after GeDi begins execution. Emfilter maps input tumour reads to the reference genome with bowtie2 [7] and outputs reads that fail to align with an exact match, i.e reads with no mismatches to the reference. Exact matching reads are discarded. We found approximately 70% of tumour reads are exact matches in our simulated datasets. Since this simulated data is built with a tool that utilizes an emperical error profile [8], we anticipate this percentage to be similar in real data. Consequently, emfilter reduces the tumour dataset to the set of tumour reads covering germline variants, somatic variants, and sequencing errors. Once emfilter has been applied, the control and filtered tumour reads provide input for suffix array construction in the following stage. Since the space and time complexity of suffix array construction increases with input text size, emfilter’s reduction of the input tumour reads reduces the runtime- and memory-resource requirements of suffix array constructing in GeDi, and ultimately, GeDi itself. Although emfilter uses alignment, GeDi is a suffix array-based SNV caller and therefore does not use the mapping coordinates produced by emfilter to detect SNVs. Accordingly, emfilter does not induce the reduced sensitivity for SNV detection at complex variant loci that afflicts reference-based SNV callers (shown in Additional file 1: Figure S7). Consequently, GeDi can detect SNVs reference-free and mapping-free (Additional file 1: Figure S7). “Runtime and memory evaluation.” section shows the reduction in resource utilisation gained by applying emfilter. We leave the application of emfilter optional to the user at the cost of increase runtime and memory usage.

After emfilter has been applied, the remaining reads are split at ‘N‘ characters and all ‘N‘-less strings of length ≥ 30 are kept for downstream processing. This completes the preprocessing stage of GeDi.

### Suffix array-based SNV detection

Next, GeDi detects SNVs using a novel suffix array-based approach that utilises two suffix arrays; in contrast to the single-suffix-array-based approach developed in SMuFin (Fig. 2b).

Suffix array-based SNV detection in GeDi proceeds as follows: GeDi constructs a primary suffix array from the preprocessed reads, and then searches for sections enriched for tumour-read-derived suffixes. Once found, GeDi then extracts tumour reads that contain suffixes within enriched sections. Like SMuFin a section is considered enriched for tumour-read-derived suffixes if two conditions are satisfied:

Firstly, identically to SMuFin, a section is considered to be enriched if the proportion of such suffixes within a section is greater than *e*_*cont*; *e*_*cont* has a default value of 0 in GeDi. Secondly, unlike SMuFin and crucial to GeDi’s sensitivity, the number of tumour-read-derived suffixes within a section must be at least *pMSS*=2; rather than the value of *MSS*=4 used in SMuFin. *pMSS*’s default value is 2. This relaxation enables GeDi to detect rare SNVs (≤ 5% allele frequency) with high sensitivity. Once tumour reads are extracted, GeDi constructs a second, auxiliary suffix array from the extracted tumour reads and their reverse complements; hence, the auxiliary suffix array consists entirely of tumour-read-derived suffixes. This auxiliary suffix array is searched for sections with size ≥*aMSS*=4; *aMSS*’s default value is four. Once these sections are identified, tumour reads containing suffixes with these sections are extracted and organised into *variant block*s: a group of reads aligned to one another that cover a genomic location containing one or more SNVs (Additional file 1: Figure S1). Note that the local alignment of reads in a variant block can be determined directly from their suffix’s offsets in the auxiliary suffix array. Construction of variant blocks completes suffix array-based SNV detection in GeDi.

The motivation for GeDi’s dual suffix array design is the overly-restrictive *MSS*=4 constraint used by SMuFin to avoid extraction of reads containing sequencing errors when searching for tumour-suffix enriched sections. This constraint can render SNVs undetectable despite sufficient coverage: Namely, when the SNVs sum total coverage is ≥*MSS* for both DNA strands, but <*MSS* on each strand. In GeDi, by relaxing this constraint to *pMSS* such SNVs remain detectable: reads covering these SNVs in both orientations will be extracted from the primary suffix array, and grouped together into a single section in the auxiliary suffix array due to the inclusion of reverse complements (Additional file 1: Figure S1 provides a graphical example). Since, GeDi reintroduces the same constraint as SMuFin on its auxiliary suffix array (*aMSS*=*MSS*=4), extraction of reads harbouring sequencing errors are still avoided. We note that, an alternative solution to a dual suffix array design is to construct a single suffix array from each input read and its reverse complement. However, due to the space complexity of suffix array construction and size of tumour-normal paired NGS datasets, such an approach would be very costly with respect to memory resource usage; which is already at a problematic scale in SMuFin. In contrast, the dual suffix array design implemented in GeDi solves the problem of detecting SNVs with a sum total coverage ≥*MSS* for both DNA strands but <*MSS* on each strand whilst simultaneously reducing extraction of reads containing sequencing errors and avoiding increasing the memory requirement of primary suffix array construction. “GeDi can detect SNVs at allelic frequencies of <1%” section provides evidence of GeDi’s dual-suffix array design retaining high sensitivity and precision for rare SNV detection with respect to SMuFin’s single-suffix array design; SNVs with a sum total coverage ≥*MSS* for both DNA strands but <*MSS* on each strand typically have low allele frequency.

### Consensus sequence construction

After suffix array-based SNV detection, GeDi holds a set of variant blocks. Since the auxiliary suffix array contains the multiple suffixes of each input read, many extracted variant blocks contain identical - and therefore redundant - information. Accordingly, to remove unnecessary downstream computation GeDi removes redundant variant blocks, keeping only one variant block from a set of blocks that each contain the same reads.

Once redundant variant blocks are removed, for each remaining variant block GeDi now constructs a (*T*,*C*)-pair Fig. (2c); a pair of consensus sequences, one derived from tumour-derived reads, *T*, the other from control-derived reads, *C*. The following method is used to construct a (*T*,*C*)-pair from a variant block: First we consider each variant block as an alignment of reads, *t*; recall that a variant block is group of locally-aligned tumour reads covering the same genomic location. *T* is constructed directly from the alignment *t* as a phred-filtered consensus string (the method to construct a phred-filtered consensus string is described below). Once *T* is constructed, the primary suffix array is then searched to identify control-read derived suffixes that cover the same genomic location as *T*; all control-read derived suffixes that share a 30-character exact match with *T* are considered to cover the same genomic location as *T*. Once such suffixes are identified, the control-tissue derived reads they belong to are arranged into an alignment *c*. Similar to the variant block alignment *t*, the local alignment of the control-tissue derived reads is computed from their suffix’s offset in the primary suffix array. Alignment *c* is then used to construct *C* as a phred-filtered consensus string. The construction of *C* completes the (*T*,*C*)-pair.

*T* and *C* are constructed from alignments *t* and *c* respectively as phred-filtered consensus strings. The method of construction is now described for a general alignment of reads *a* and phred-filtered consensus string *S*.

Let the number of columns in *a* from the start of left-most read to the end of the right-most, i.e the length of *a*, be |*a*|, where *a*_*j*_,1≤*j*≤|*a*|, is the *j*th column of *a*. Using *a*, we compute frequency matrix $\boldsymbol {F^{a}} \in \mathbb {N}_{0}^{|\Sigma | \times |a|}$, where *Σ*={*A*,*C*,*G*,*T*},|*Σ*|=4. The *j*th column of ***F***^***a***^ is ***F***^***a***^_∗,*j*_=(*f*_*A*,*j*_,*f*_*C*,*j*_,*f*_*G*,*j*_,*f*_*T*,*j*_)^⊤^, where each entry describes the count of A, C, G, T characters in column *a*_*j*_ with a phred score ≥*p* respectively; *p*’s default value is 35. Using ***F***^***a***^, *S* is constructed as the string $S_{j} = consensus\left (\boldsymbol {F^{a}_{*,j}}\right)$, where *S*_*j*_∈*Σ* is the *j*th symbol in *S*. $consensus\left (\boldsymbol {F^{a}_{*,j}}\right)$ identifies the numerically largest entry in ***F***^***a***^_∗,*j*_, denoted *f*_*σ*,*j*_,*σ*∈*Σ*, and returns symbol *σ*. Counting in ***F***^***a***^ only the characters with phred score ≥*p* stops characters with phred score <*p* from contributing towards the consensus sequence, *S*. This mitigates the propagation of sequencing errors into *S*, and ultimately mitigates false positive SNV calls. Note that, ties for the most frequent base at some column *j* of ***F***^***a***^ are resolved by picking the most frequent base (amongst the tied bases) with any phred score. If a tie still remains, the lexicographically smallest base is chosen for *S*_*j*_.

Consensus sequence construction ends when a (*T*,*C*)-pair is computed for each variant block.

### Consensus sequence filtering

In the following SNV calling stage, GeDi will examine aligned *T*-*C* pairs and call single character mismatches as SNVs. Accordingly, removal of false positives from these pairs is critical to increasing GeDi’s precision. Although many potential sequencing error-based false positives will have been removed during *T*-*C* pair construction, we found three genomic features were commonly associated with false positive calls. We therefore developed three filters designed to remove false positives arising from each of these features. Each filter is applied after *T*-*C* pair construction (Fig. 2c).

Our first filter, *indel filter*, removes false positive SNV calls caused by somatic indels. A complete description of this filter and its effect is given in Additional file 1: Method 3.

Our second filter, *masking filter*, was designed to reduce false positives caused by SNPs. Using the previous section’s notation, SNPs cause false positives when the most frequent allele differs across *t* and *c* alignments. This difference causes a sequence mismatch at the SNP site which GeDi then reports as a false positive (Additional file 1: Figure S2). To tackle this issue, we leverage the property that the column of *c* containing the SNP, *c*_*snp*_, is likely to contain both SNP alleles. Accordingly, in *c*’s frequency matrix column ***F***^***c***^_∗,*snp*_ will contain more than one non-zero entry. Let indicator function $\mathbbm {1}(k)$ return 1 if *k*>*e*, 0 otherwise; *e*’s default value is 0.1. We define *masking filter* as follows: For 1≤*j*≤|*t*|, replace *T*_*j*_ with *C*_*j*_ if:

$${\sum\nolimits}_{\sigma \in \{A,T,C,G\}}\mathbbm{1}(r_{\sigma,j}) \quad > \quad 1, $$

where,

$$r_{\sigma,j} = \frac{f_{\sigma,j}}{sum\left(\boldsymbol{F_{*,j}}\right)}, $$

and *sum*(***F***_***∗,j***_) is the sum of the entry in ***F***_***∗,j***_. Accordingly at sites likely to contain SNPs, *T* characters are replaced (masked) with *C* characters (Additional file 1: Figure S2 provides a graphical explanation). Parameter *e* in $\mathbbm {1}(n)$ reduces sensitivity loss that arises from masking consensus sequence positions containing sequencing errors and SNVs in the same alignment column.

Our third filter, *multi-locus filter*, was designed to reduce false positives caused by exact repeat genome sequences. *T*-*C* pairs covering such sequences are often invalid, since the *t* and *c* alignments used to construct them contain reads from multiple genome locations. To tackle this issue, multi-locus filter identifies and discards these invalid pairs by exploiting the property that their ***F***^***c***^ and ***F***^***t***^ matrices often contain multiple columns with >1 non-zero entry: A consequence of the reads of alignment *c* or *t* deriving from multiple locations is that, beyond the exact matching repeat, their sequences diverge. Consequently, a column *j* of ***F***^***c***^ or ***F***^***t***^ that represents a position *c* or *t* outside the exact matching repeat is likely to contain more than one entry with a large quantity; due to the presence of multiple different bases in the alignment column. Formally, an entry, *f*_*σ*,*j*_, has a large quantity if it has $\mathbbm {1}(r_{\sigma,j}) = 1$ for its corresponding *r*_*σ*,*j*_, and thus induces a masking event. Accordingly, if a *T*-*C* pair’s frequency matrices induce ≥5 masking events (we found five allows for some SNPs and sequencing errors to be present without discarding) multi-locus filter will discard the pair. By doing so, invalid pairs containing information from multiple genomic locations are discarded. Additional file 1: Table S2 shows the dramatic reduction in false positives reported by GeDi when masking and multi-locus filter are applied.

After application of all filters to each *T*-*C* pair, consensus sequence filtering is complete.

### SNV calling

After consensus sequence filtering, GeDi calls SNVs, determining and reporting each detect SNV’s genome coordinate and structure back to the user (Fig. 2d). To achieve this for a given consensus pair, the control consensus sequence is mapped to the human reference genome using Bowtie2. The chromosome to which the control sequence aligns and mapping coordinate, *m*, is recorded. The tumour and control consensus sequences are then aligned to one another and examined. Any single character mismatches - now assumed to be genuine SNVs - are identified. Each SNV’s index within the tumour consensus sequence, *i*, is recorded along with its control and tumour variant bases. Each SNV’s chromosome coordinate, *μ*, is then calculated using formula *μ*=*m*+*i*. Finally, GeDi calls each SNV, reporting the chromosome in which the SNV is located, *μ*, the control base, and the tumour variant base to the user. GeDi performs this calling process for each consensus sequence pair and subsequently terminates.

## Results

Additional file 2 (raw_data.zip) contains raw data for generating the figures and tables presented in this section and presented in Additional file 1 (SupplementaryData.pdf).

### GeDi can detect SNVs at allelic frequencies of <1%

SNVs present with allele frequency of ≤ 5% (rare SNVs) occur frequently in genes commonly mutated during cancer (for example, EGFR, KRAS, PIK3CA, and BRAF), and can provide insight into the dynamics of subclonal tumour populations. Hence, accurate detection of rare SNVs has important applications both clinically and fundamentally [9]. GeDi’s dual suffix array design enables reference-free detection of rare SNVs with high precision. To evaluate GeDi’s design for rare SNV detection, we generated simulated targeted deep-sequencing datasets and analysed these datasets with GeDi and MuTect. To generate these datasets, we selected five random 1 Mbp target sequences from hg19 chromosomes 1, 8, 9, 15 and 22, and for each target sequence, used ART NGS simulator [8] to generate seven tumour-control paired targeted deep-sequencing datasets with 1000x coverage, 200 SNVs, and each with a different average allele frequency of either 50%, 25%, 10%, 5%, 2%, 1% or <1% (Additional file 1: Method 1 provides a detailed description of dataset construction).

**Fig. 3:**
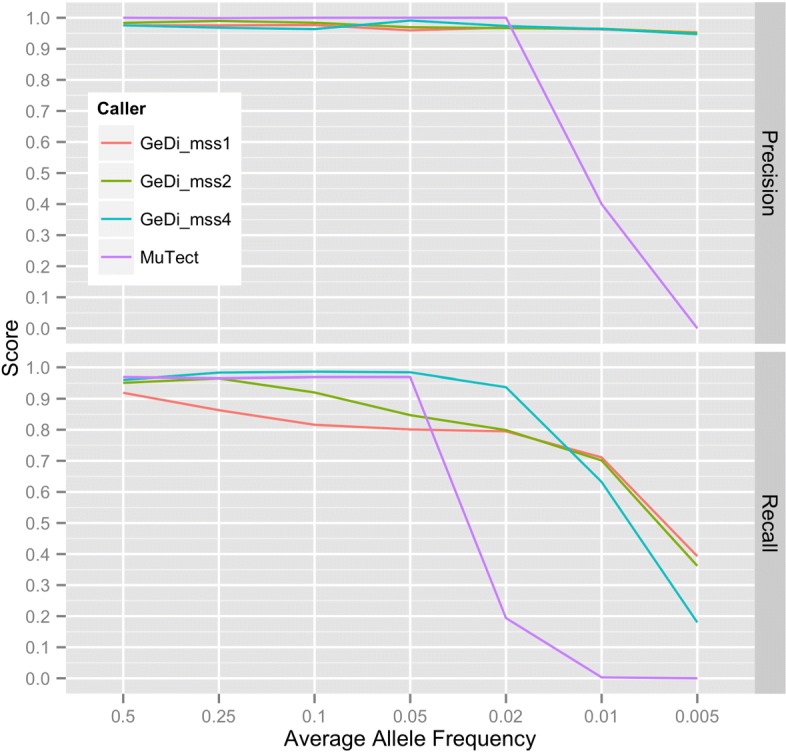
Precision and recall of GeDi and MuTect for SNV detection at decreasing allele frequencies. We analysed the 35 simulated tumour-control paired targeted deep-sequencing datasets with MuTect, once using default settings, and GeDi, three times each time setting *pMSS* to 1, 2 and 4; in the legend, GeDi_mss1, GeDi_mss2, GeDi_mss4 refer respectively to the three GeDi analyses. For each analysis we then calculated the precision and recall attained by MuTect and GeDi. For each of the seven average allele frequencies, the arithmetic mean of the precision and recall attained by MuTect and GeDi (in each mode) across the five datasets (generated from the target sequences of hg19 chromosomes 1, 8, 9, 15, 22) are shown

Figure 3 shows the average precision and recall attained by GeDi and MuTect for these datasets in response to average allele frequency. Note that, in order to determine the effect of GeDi’s relaxed *pMSS* constraint in its dual suffix array design, we ran GeDi three times, setting *pMSS* to four, two and one for each run. For input into MuTect, bowtie2 was used to generate SAM files (Additional file 1: Command 5 shows an example command) and Picard Tools (http://broadinstitute.github.io/picard/) was used to convert SAM files to bam [7]. We ran mutect with default parameters. When *pMSS*=4, suffix array-based SNV detection in GeDi emulates the restrictive *MSS* constraint applied in SMuFin’s approach. As expected, GeDi displays greater recall for SNV detection at very low allele frequencies (≤ 1%) when *pMSS* is relaxed to one or two rather than four (Fig. 3), highlighting the superiority of GeDi’s dual suffix array design for low frequency SNV detection compared to SMuFin’s single suffix array approach. Furthermore, as *aMSS*=4 in all runs, despite recall increasing when *pMSS* is relaxed, GeDi’s precision remains high (95%). Interestingly, we found that GeDi has higher recall when detecting SNVs at ≥ 2% when *pMSS*=4 or 2 rather than 1. We anticipate this is due to multi-locus filter discarding additional variant blocks under the *pMSS*=1 or 2 regime: Variant blocks containing reads derived from one DNA strand only under a *pMSS*=4 regime can contain reads derived from the opposite DNA strand in the *pMSS*=1 or 2 regime. When applying the masking filter to such blocks, the additional reads can increase the number of masking events if they contain sequencing errors. This can trigger the multi-locus filter (applied when the number of masking events increases above a threshold; see Implementation).

In stark contrast to GeDi, MuTect is incapable of SNV detection at allele frequencies ≤ 1% and exhibits a large drop in recall at 2% (Fig. 3), suggesting GeDi will outperform MuTect for SNV detection at very low allele frequencies (2%) on real datasets. GeDi’s ability to detect SNVs at lower allele frequencies than MuTect is a consequence of the different minimum number of tumour-derived reads required to call SNVs between the two algorithms: GeDi requires at least *aMSS*=4 tumour-derived reads that support the non-reference allele (with a phred score of ≥*p*=35), whilst MuTect requires 14 non-reference-allele supporting tumour-derived reads (https://software.broadinstitute.org/cancer/cga/mutect).

Given GeDi’s potential to detect SNVs at allele frequencies far below MuTect, we analysed two real paired tumour-blood targeted deep sequencing datasets targeting loci on chromosome 17 and chromosome 22 with GeDi; datasets TSD:chr22 and TSD:chr17 respectively (data currently unpublished). These datasets were derived from a fresh-frozen specimen appertaining to a female ERa+ breast cancer patient treated 2 years with Aromatase Inhibitors. We compared GeDi’s output to MuTect’s; methods describing the preprocessing of this data and MuTect analysis are given in Additional file 1: Method 5.

We ran GeDi in its default mode (*pMSS*=2). For TSD:chr22, MuTect made a total of nine SNV calls. GeDi made all nine of the SNV calls made by MuTect, plus four additional calls not made by MuTect (Table 1). We inspected these four additional calls made by GeDi in IGV (http://www.broadinstitute.org/igv). All four occurred with a low allele frequency (range of 3.9% to 0.9%); for the four calls, Additional file 1: Figures S3–6 shows IGV snapshots, whilst Additional file 1: Table S3 provides allele frequencies and coverage counts. The above results, the low allele frequency of the four calls, and GeDi’s stringent criteria for SNV calling (at least *aMSS* reads with a phred score ≥ 35 must contain the variant allele) suggest these additional calls are genuine SNVs undetected by MuTect. Analogous results were found when analysing TSD:chr17: MuTect made a total of 40 SNV calls. GeDi called 36 of these, plus an additional 37 (Table 1). Since Fig. 3 suggested GeDi’s recall for SNVs with allele frequency closer to 50% is greater when *pMSS*=4, we took the union of GeDi’s output when run with *pMSS*=1 and 4 to determine if the intersection between the calls made by GeDi and MuTect could be increased. Additional file 1: Table S4 provides the output from this analysis, and shows the results are very similar to those given in Table 1, where *pMSS*=2; the intersection increased by a single SNV.

**Table 1:** Number of GeDi and Mutect SNV calls for datasets TSD:chr22, TSD:chr17

Dataset	Mutect calls	GeDi calls	Intersection
TSD:chr22	9	13	9
TSD:chr17	40	73	36

Intersection describes the number of SNV calls MuTect and GeDi made in common

### Somatic short range SNV cluster (sSRSC) detection

A major issue with reference-based SNV callers is the reduced sensitivity they exhibit at complex variant loci due to incorrect mapping coordinates [2]. Since GeDi is a suffix array-based SNV caller, it does not rely on mapping coordinates to detect SNVs, and therefore, should retain sensitivity for SNV detection at complex variant loci.

Short Range SNP clusters (SRSC) are variant genomic features formally defined as a genomic loci containing *k* SNPs within window length *W*=100 [1]. SRSC have been shown to induce incorrect mapping coordinates for reads covering them. Hence, reference-based variant callers are likely to show insensitivity for SRSC detection [1]. We define a somatic version of SRSC, the *somatic Short Range SNV Cluster* (sSRSC), as genomic loci containing *k*≥2 SNVs within window length *W*=100 occurring in tumour genomes; we define, k-sSRSC as a sSRSC containing *k* SNVs. Accordingly, sSRSC are a formalised subset somatic clustered hypermutation.

As sSRSC are identical to SRSC apart from their occurrence in tumour genomes, they too will induce incorrect mapping coordinates, and are therefore ideal features to evaluate GeDi’s sensitivity for SNV detection at complex variant loci. We generated five 30x simulated datasets containing 500 sSRSC of size 2≤*k*≤20 of hg19 chromosomes 1, 8, 15, 17 and 22 (randomly chosen) and calculated GeDi’s and MuTect’s precision and recall when analysing these datasets. Our chosen size distribution 2≤*k*≤20 reflects the known size distribution of SRSC within the human genome [1]. As were not testing the effect of allele frequency, average allele frequency kept at 50% for all datasets. A full explanation of how these datasets were generated is given in Additional file 1: Method 2.

**Fig. 4:**
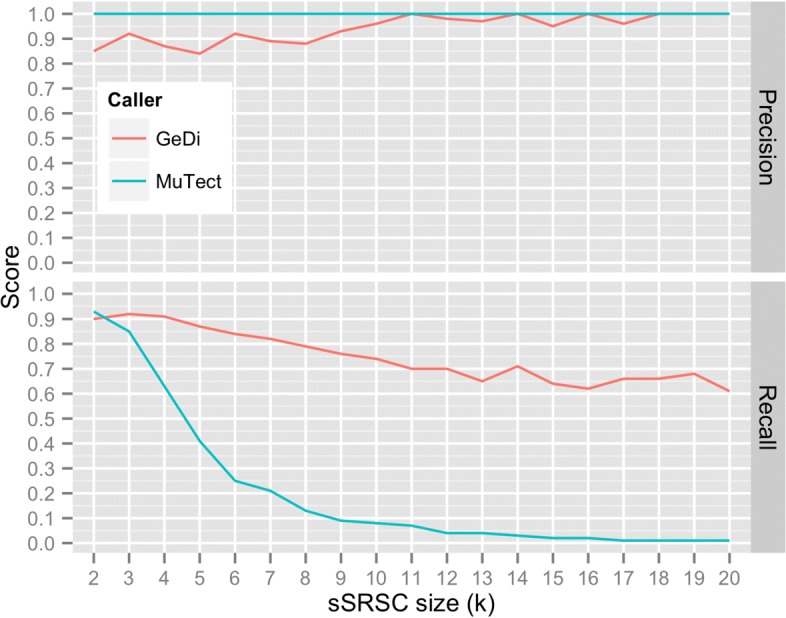
Precision and recall of GeDi and MuTect for sSRSC detection

Figure 4 shows the *k-precision* and *k-recall* attained by GeDi and MuTect for sSRSC as *k* increases. We define $k-recall= \frac {k-hits}{k-total}$, where *k*−*hits* is the number of detected SNVs residing in all k-sSRSC across all five datasets, and *k*−*total* is the total number of SNVs residing in all k-sSRSC across all five datasets. We define $k-precision = \frac {k-hits}{k-fp + k-hits}$, where *k*−*fp* is the total number of false positive calls made within all genuine k-sSRSC added to the number of SNVs within reported k-sSRSCs that contain entirely false positives.

As expected, GeDi shows superior recall for SNV detection at sSRSC than the reference-based SNV caller MuTect. For sSRSC of size *k*≥3, GeDi’s *k-recall* outperforms MuTect’s for sSRSC detection; indeed, for sizes *k*≥9, MuTect’s *k-recall* drops below 10%, whilst GeDi’s remains above 60%. Furthermore, we find that as *k* increases, GeDi’s *k-precision* increases, making GeDi very precise at detecting large sSRSC (Fig. 4).

### Whole genome sequencing evaluation

Dataset EGAD00001001859 is a high average coverage (272x control, 314x tumour) medulloblastoma tumour-control paired WGS dataset. It has an associated set of 1255 curated SNV calls (Gold Set), generated by analysing all sequence data from EGAD00001001859 (which we hereafter call MB) with six different popular SNV calling pipelines and accepting calls made by at least three [5]. Accordingly, Golden Set’s SNVs are stereotypical what can be identified by current SNV calling pipelines at high coverage. MB contains a data subset, MB:L.A, with average coverage more typical of standard WGS datasets (29.6x control, 40.5x tumour). Combined with Gold Set, MB:L.A can be used to benchmark SNV callers [5].

To evaluate GeDi’s performance on WGS datasets, we analysed MB:L.A with GeDi when running in default mode (*pMSS*=2) and with *pMSS*=4. To ensure fair comparison with the SNV callers evaluated in [5], prior to analysis of MB:L.A with GeDi we preprocessed the raw sequencing reads of MB:L.A exactly following practices given in [5]; practices, such as filtering and quality control, are described in Additional file 1: Method 6. We calculated the precision and recall attained by GeDi against Gold Set when analysing MB:L.A in the two aforementioned modes (Table 2, row one and two respectively). In default mode, at 66%, GeDi’s recall against Gold Set ranks above six of the 18 SNV calling pipelines benchmarked in [5] including SMuFin. Although the recall achieved by GeDi is lower than we anticipated, it is only 10% below the most sensitive pipeline benchmarked in [5]. Furthermore, we find when run GeDi with *pMSS*=4, GeDi’s recall decreased significantly, and is almost identical to that achieved by SMuFin [5]. This further highlights the increased sensitivity afforded by GeDi’s dual suffix array design relative to SMuFin’s design.

**Table 2:** Precision, Recall and F-Score attained by GeDi when analysing dataset MB:L.A

pMSS	Total calls	Precision	Recall	F-Score
2	8118	0.10	0.66	0.18
4	6519	0.11	0.58	0.19

When analysing MB:L.A in default mode, 7289 of the 8118 SNV calls made by GeDi were not present within Gold Set; we refer to these SNV calls as (*non-Gold Set SNV calls*). Because of these calls, GeDi’s precision against Gold Set ranks lowest out of the 18 tested pipelines [5]. To determine whether non-Gold Set SNV calls contain biological signal, we calculated the percentage of these calls occurring within known cis-regulatory or transcribed regions (See Additional file 1: Method 7 for details on how these percentages were computed). Since these regions are functional genomic regions, if non-Gold Set calls contain biological signal, we expect the percentage of non-Gold Set calls and Gold Set calls occurring in these regions to be similar; as Gold Set calls are high-quality curated calls and should therefore exhibit genuine biological signal. We found 55% of non-Gold Set SNVs occur in such regions. In contrast, only 51% of the 1255 SNV calls comprising Gold Set occur in such regions (Table 3). Hence, our result demonstrates that non-Gold Set SNV calls show a bias towards cis-regulatory or transcribed regions even stronger than the bias in Gold Set. Therefore, given the acceptance of Gold Set as high quality, although non-Gold Set SNV calls remain putative, this result suggests non-Gold Set SNV calls are enriched for genuine biological signals (i.e real SNVs).

**Table 3:** Percentage of SNVs located in transcribed or functional regions for Gold Set, all 8118 SNV calls output by GeDi in default mode (All GeDi), 7289 non-Gold Set SNV calls, and 3331 sSRSC-residing SNV calls (sSRSC-residing)

Set	Percent
Gold set	51
All GeDi	54
Non-Gold set	55
sSRSC-residing	54

Percents rounded to nearest whole percent

**Fig. 5:**
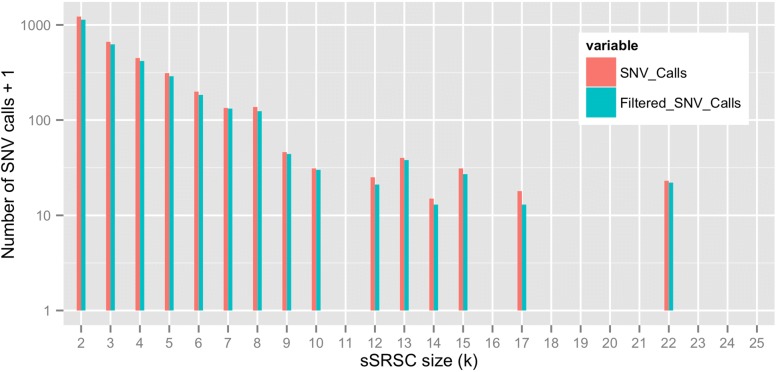
Frequency distribution of sSRSC identified by GeDi when analysing dataset MB:L.A in default mode. Once binned, we removed SNP calls by filtering against http://hgdownload.cse.ucsc.edu/goldenpath/hg19/database/ snp150.txt.gz. The y-axis is presented in log-scale. Accordingly, 1 has been added to each sSRSC bin to avoid log(0), i.e undefined values, for bins which no sSRSC of that size were detected. Hence "Number of SNV calls +1" is presented on the y-axis label

Given GeDi’s high sensitivity for sSRSC detection, we used GeDi to characterise sSRSC within MB:L.A. 3331 of 8118 SNV calls made by GeDi when analysing MB:L.A in default mode occurred in sSRSCs (*sSRSC-residing SNV calls*). The overwhelming majority of these were non-Gold Set SNVs (only 16 out of 3331 were present in Gold Set) and are therefore almost entirely absent from Gold Set. Figure 5 shows the absolute frequency of sSRSC-residing SNV calls binned by their occurrence in sSRSC of size *k* (red bars), and with SNPs filtered out (blue bars). We found no sSRSC of size *k*>25. Furthermore, comparison of blue and red bars show that some sSRSCs identified by GeDi contain known SNPs amongst the SNV calls. 54% percent of the sSRSC-residing SNV calls occur within known cis-regulatory or transcribed regions, which like the total non-Gold Set SNVs, is above the percent attained by Gold Set and is suggestive that these putative calls are genuine SNVs (Table 3).

### Runtime and memory evaluation.

SMuFin suffers from large runtime and memory resource requirements. In GeDi, emfilter was designed to combat these issues, reducing both runtime and memory requirements for suffix array construction. Beyond emfilter, we made extensive use of OpenMP [10] to further reduce GeDi’s runtime, parallelising GeDi’s suffix array-based SNV detection, consensus sequence construction and filtering stages. Table 4 row one shows the runtime and memory requirements of GeDi, MuTect and SMuFin when analysing a simulated 30x dataset of hg19 chromosome 22 (dataset SMuFin:chr22) downloaded from the SMuFin website (http://cg.bsc.es/smufin/); despite significant effort we were unable to get SMuFin to execute successfully on any other datasets. Results show that GeDi’s runtime and memory resource requirements are significantly reduced by emfilter, and both memory and runtime resource requirements of GeDi are well below those of MuTect and SMuFin.

**Table 4:** Runtime and memory (rss) evaluation of gedi, smufin and mutect

Dataset	Size (Million reads / GB)	RSS (GB)			Runtime (hours:minutes)		
		GeDi (no emfilter)	MuTect	SMuFin (256 thread)	GeDi (no emfilter)	MuTect	SMuFin (256 thread)
SMuFin:chr22	26 / 4.40	16 (19)	67	22 (107)	0:20 (0:30)	1:33	17:48 (3:47)
TSD:chr17	11 / 2.43	7	67	-	0:06	0:50	-
TSD:chr22	4 / 0.87	3	108	-	0:02	0:38	-
MB:L.A	2052 / 69.21	1017	97*	-	71:39	1800*	-

no emfilter (bracketed values for gedi) shows gedi’s resource requirements when emfilter is off, for all other gedi runs, emfilter is on. 256 thread (bracketed values for smufin) shows smufin’s resource requirements when run with suggested command at http://cg.bsc.es/smufin/, whilst values without brackets show SMuFin’s requirements when run with 32 logical threads. GeDi and MuTect were always run with 32 logical threads, apart from analysis of MB:L.A where 64 logical threads were used for both callers. A full description of the methods used to perform this benchmark are provided in Additional file 1: Method 4. All analyses were performed on the same computing system with Xeon: E5-4650v2 CPUs. Runtime and RSS was recorded using GNU Time 1.7 (https://www.gnu.org/), where runtime is the elapsed wall clock time and RSS is the maximum residency set size. Asterisk values: MuTect took 72 hours (maximum user runtime) on our system to analyse 4% of the human genome (percentage along genome is given in MuTect output). Accordingly, assuming uniform coverage of MB:L.A data across the genome, we estimated MuTect’s runtime in hours for analysis of the complete MB:L.A dataset by multiplying 72 by 25

Table 4 rows 2–4 show that GeDi’s runtime and memory requirements are well below those of MuTect when analysing targeted deep-sequencing datasets making GeDi a very practical algorithm for such analyses. For 30x WGS datasets however, GeDi’s memory resource requirements are large (Table 4 row 5), well above MuTect’s. This is due to the large memory resources required during GeDi’s primary suffix array construction. Accordingly, GeDi requires a machine with large memory resources when analysing WGS datasets.

## Discussion

In this work we introduce GeDi, a suffix array-based SNV caller capable of detecting SNVs reference-free and mapping-free. GeDi’s design enables high sensitivity SNV detection at complex variant loci such as sSRSC, a subset of somatic clustered hypermutation. We found this in stark contrast to MuTect, a reference-based SNV caller, which exhibits greatly reduced sensitivity at such loci. Accordingly, GeDi shows great potential for characterising SNVs at complex variant loci in tumour genomes and will likely outperform other reference-based SNV callers. Indeed, GeDi detected a large number of putative SNVs occurring within complex variant loci (sSRSC) of dataset MB:L.A, providing previously unreported insight into somatic clustered hypermutation within tumour genomes.

Not only is GeDi capable of detecting SNVs at complex variant loci, it is also capable of detecting rare SNVs whilst maintaining high precision. This feature of GeDi is well-suited towards targeted deep sequencing analyses, where detection of rare SNVs is a primary goal. Indeed, when analysing a targeted deep sequencing dataset (TSD:chr22) GeDi found four previously undetected putative SNVs. Each of these SNVs occurred at very low allele frequency (<0.04), which likely rendered them undetectable by the previously utilised SNV caller (MuTect). Given GeDi’s potential for rare SNV detection and its very practical resource requirements, we expect GeDi to find fruitful application in targeted deep sequencing analyses.

GeDi advances the suffix array-based approach to SNV calling beyond the original approach outlined in SMuFin [3]: Its dual suffix array design resolves SMuFin’s insensitivity at low allele frequencies; its use of a preprocessing filter to reduce resource requirements during suffix array construction and its highly parallelised architecture resolve SMuFin’s large memory and runtime issues; and it performs favourably on both WGS and targeted deep sequencing datasets, outperforming SMuFin for sensitivity. However, future work still remains. First, GeDi’s significant memory resource requirement when analysing large WGS datasets motivates further work into memory reduction. Second, since GeDi can detect SNVs outside the realm of reference-based SNV callers, work is needed to accurately determine GeDi’s precision on real datasets by validation with Sanger sequencing. Third, if shown necessary through Sanger sequencing validation, GeDi’s SNV classification method could be improved by integration of statistical or deep-learning methods [6, 11]. Fourth, GeDi can be readily extended to detect short (< read length) indel variants as both short indels and SNVs will induce sections enriched for tumour-read-derived suffixes with the primary suffix array. Extending GeDi to detect indels reference-free will provide a method for sensitive indel calling in complex variant regions that may outperform reference-based indel callers in these regions. Finally, coloured De Bruijn graph-based variant callers (bubble callers) also detect SNVs reference-free and mapping-free [12]. However, bubble callers often rely on simple graph structure (such as the 2k + 2 bubble) to detect SNVs. As a result, bubble callers often fail to detect SNVs that induce more complex graph structures [13]. Since GeDi does not rely on graph topology to detect SNVs, in addition to reference-based SNV callers, it may outperform many bubble callers at complex variant loci. Therefore, a comparison of GeDi’s performance against relevant bubble callers will be of value.

## Conclusions

Despite the advance of reference-based SNV callers, these algorithms are prone to reduced sensitivity at complex variant loci where incorrect read mapping is common. It is therefore likely that many complex variant loci remain vastly under-characterised within tumour genomes. Our work here shows that GeDi’s detection of SNVs without reliance on mapping coordinates has the capacity to increase the repertoire of detectable SNVs within tumour genomes by enabling characterisation of SNVs at complex variant loci with high sensitivity. Accordingly, we expect GeDi to uncover novel biological insights previously undetected by the cancer genomics research community.

## Availability and requirements

- Project name: GeDi
- Project home page: https://github.com/izaak-coleman/GeDi
- Operating system: Platform Independent.
- Programming language: C++
- Other requirements: g++ 4.8.1 or greater, cmake (https://cmake.org/), and boost (https://www.boost.org/) must be installed.
- License: MIT License

## Supplementary information

**Additional file 1** SupplementaryData.pdf is available online, and contains all additional data referenced in the main text.

**Additional file 2** raw_data.zip is available online and contains raw data for generating the figures and tables presented in the main text and SupplementaryData.pdf.

## Data Availability

Source code and example data files to run GeDi can be found at https://github.com/izaak-coleman/GeDi. Our work makes use of two unpublished tumour-control paired targeted sequencing datasets referenced as TSD:chr22 and TSD:chr17 throughout this work Additional file 1. These sequencing datasets were made available to reviewers during the peer-review process. All other raw data files (including the variant call files output from GeDi when analysing TSD:chr22 and TSD:chr17) are publicly on BMC Bioinformatics.

## Abbreviations

aGSA: Auxiliary generalised suffix array
GeDi: Generalised suffix array-based Direct SNV caller
NGS: Next generation sequencing
pGSA: Primary generalised suffix array
SMuFin: Somatic mutation finder [3]
SNP: Single nucleotide polymorphism
SNV: Single nucleotide variant
sSRSC: Somatic short range SNV cluster
WGS: Whole genome sequencing

## References

[1] Boenn M (2018). Shrangesim: Simulation of single nucleotide polymorphism clusters in next-generation sequencing data. J Comput Biol.

[2] Degner JF, Marioni JC, Pai AA, Pickrell JK, Nkadori E, Gilad Y, Pritchard JK (2009). Effect of read-mapping biases on detecting allele-specific expression from rna-sequencing data. Bioinformatics.

[3] Moncunill V, Gonzalez S, Bea S, Andrieux LO, Salaverria I, Royo C, Martinez L, Puiggros M, Segura-Wang M, Stuetz AM, Navarro A, Royo R, Gelpi JL, Gut IG, Lopez-Otin C, Orozco M, Korbel J, Campo E, Puente XS, Torrents D (2014). Comprehensive characterization of complex structural variations in cancer by directly comparing genome sequence reads. Nat Biotechnol.

[4] Yamagata Koichi, Yamanishi Ayako, Kokubu Chikara, Takeda Junji, Sese Jun (2016). COSMOS: accurate detection of somatic structural variations through asymmetric comparison between tumor and normal samples. Nucleic Acids Research.

[5] Tyler SA (2015). A comprehensive assessment of somatic mutation detection in cancer using whole-genome sequencing. Nat Commun.

[6] Cibulskis K, Lawrence MS, Carter SL, Sivachenko A, Jaffe D, Sougnez C, Gabriel S, Meyerson M, Lander ES, Getz G (2013). Sensitive detection of somatic point mutations in impure and heterogeneous cancer samples. Nat Biotechnol.

[7] Langmead B, Salzberg SL (2012). Fast gapped-read alignment with bowtie 2. Nat Methods.

[8] Huang W, Li L, Myers JR, Marth GT (2012). Art: a next-generation sequencing read simulator. Bioinformatics.

[9] Shin H-T, Choi Y-L, Yun JW, Kim NKD, Kim S-Y, Jeon HJ, Nam J-Y, Lee C, Ryu D, Kim SC, Park K, Lee E, Bae JS, Son DS, Joung J-G, Lee J, Kim ST, Ahn M-J, Lee S-H, Ahn JS, Lee WY, Oh BY, Park YH, Lee JE, Lee KH, Kim HC, Kim K-M, Im Y-H, Park K, Park PJ, Park W-Y (2017). Prevalence and detection of low-allele-fraction variants in clinical cancer samples. Nat Commun.

[10] Dagum L, Menon R (1998). Openmp: An industry standard api for shared-memory programming. IEEE Comput Sci Eng.

[11] Poplin R, Chang P-C, Alexander D, Schwartz S, Colthurst T, Ku A, Newburger D, Dijamco J, Nguyen N, Afshar PT, Gross SS, Dorfman L, McLean CY, DePristo MA (2018). A universal SNP and small-indel variant caller using deep neural networks. Nat Biotechnol.

[12] Iqbal Z, Caccamo M, Turner I, Flicek P, McVean G (2012). De novo assembly and genotyping of variants using colored de Bruijn graphs. Nat Genet.

[13] Bateman A, Treangen TJ, Pop M (2016). Limitations of current approaches for reference-free, graph-based variant detection. Proceedings of the 7th ACM International Conference on Bioinformatics, Computational Biology, and Health Informatics. BCB ’16.

