# Supplementary material for: GeDi: applying suffix arrays to increase the repertoire of detectable SNVs in tumour genomes
Source: BMC Bioinformatics. 2020 Feb 5;21:45. doi: 10.1186/s12859-020-3367-3 (PMC7003401; doi:10.1186/s12859-020-3367-3)
Supplement: Supplementary file 1 — Additional file 1 SupplementaryData.pdf is available online, and contains all additional data referenced in the main text. [file 12859_2020_3367_MOESM1_ESM.pdf]

Supplementary Data. GeDi: applying suffix arrays to increase the repertoire of detectable SNVs in tumour genomes.

Coleman, Izaak
Department of Systems Biology, Columbia University
Department of Computing, Imperial College London

Corleone, Giacomo
Department of Surgery and Cancer, Imperial College Hammersmith

James, Arram
Department of Computing, Imperial College London

Ho-Cheung, Ng
Department of Computing, Imperial College London

Luca, Magnani
Department of Surgery and Cancer, Imperial College Hammersmith

Wayne, Luk
Department of Computing, Imperial College London

Contents

1 Methods **2**
1.1 Method 1: Generating simulated targeted deep-sequencing datasets. . . . 2
1.2 Method 2: Generating simulated sSRSC-containing datasets 2
1.3 Method 3: Removing false positives arising from somatic indels 2
1.4 Method 4: Benchmarking resource requirements 3
1.5 Method 5: Preprocessing and MuTect analysis of datasets TSD:chr17 and TSD:chr22 3
1.6 Method 6: MB:L.A preprocessing 3
1.7 Method 7: Calculation of percentage of GeDi calls within cis-regulatory or transcribed regions . . . 4

2 Figures **5**
2.1 Figure 1. Graphical explanation of GeDi's dual suffix array design. 5
2.2 Figure 2. Graphical explanation of GeDi's masking filter. 6
2.3 Figure 3-6. IGV snapshots of SNV calls made by GeDi when analyzing dataset TSD:chr22. 7
2.4 Figure 7. Effect of emfilter on recall and precision for SNV calling across different sSRSC. 10

3 Tables **10**
3.1 Effect of indel filter on false positive reduction. 10
3.2 Effect of masking filter on false positive reduction. 11
3.3 Call information of SNV calls made by GeDi when analyzing dataset TSD:chr22. 11
3.4 SNV calls made by GeDi when combining output from runs with pMSS = 1 and pMSS = 4. 11

4 Commands **11**

1 Methods

1.1 Method 1: Generating simulated targeted deep-sequencing datasets.

To evaluate GeDi for low frequency SNV detection we generated multiple simulated targeted deep-sequencing datasets using the method described in this section. We first generated two copies of hg19 chromosomes 1, 8, 9, 15 and 22 (randomly picked from ftp://ftp.broadinstitute.org/bundle/hg19/ucsc.hg19.fasta.gz) and to each copy added between 100-300 random SNPs from http://hgdownload.cse.ucsc.edu/goldenPath/hg19/database/snp147.txt.gz resulting in pairs of personalised "maternal" and "paternal" chromosome files. For each pair of chromosome files, we extracted a two 1 Mbp sequences, one from the paternal file, the other from the maternal file, both starting at the same coordinate. We call these sequences the paternal and maternal target sequences respectively. The beginning coordinate of each pair of paternal and maternal target sequences was chosen randomly apart from avoiding coordinates that lead to an overlap between the target sequences with centromeric regions (defined in http://hgdownload.cse.ucsc.edu/goldenPath/hg19/database/cytoBand.txt.gz).

To simulate SNVs, we made a copy of each maternal and paternal target sequence and added 100 randomly located SNVs to the copies. This resulted in four target sequences for a given chromosome: two *control target sequences*, the original paternal and maternal target sequences; and two *tumour target sequences*, the copied control target sequences with 100 randomly located SNVs added to each.

For each chromosome, the four target sequences were used as templates to produce seven 1000x simulated targeted-deep sequencing datasets using ART NGS dataset simulator (Huang *et al.*, 2012). Supplementary Data, Command 6 shows an example of the command and parameters used when constructing datasets with ART. The seven datasets produced for each chromosome had differing average allele frequencies of 0.50, 0.25, 0.10, 0.05, 0.02, 0.01 and 0.005. To construct, for example, the dataset with 0.005 average allele frequency, we produced the tumour data by generating two 495x coverage datasets using the paternal and maternal control target sequences as a template, and combining these with two 5x coverage datasets using the paternal and maternal tumour target sequence as a template into a single dataset. Similarly, to produce the control data, we combined two 500x coverage datasets using the paternal and maternal control target sequence as a template into a single dataset. All datasets were produced using this method and differed only in the coverages required to generate the necessary average allele frequencies. In total, this process gave 35 1000x simulated targeted deep-sequencing tumour-control paired NGS datasets: For each of the five chromosomes, seven datasets with differing average allele frequencies.

1.2 Method 2: Generating simulated sSRSC-containing datasets

To evaluate GeDi's ability to detect sSRSC we built simulated datasets containing sSRSC ranging from size $k = 2$ to $k = 20$ using ART NGS dataset simulator following the method described in this section. We first generated two copies of hg19 chromosomes 1, 8, 15, 17 and 22 (randomly picked from ftp://ftp.broadinstitute.org/bundle/hg19/ucsc.hg19.fasta.gz) and to each copy added between 100-300 random SNPs from http:// hgdownload.cse .ucsc.edu/goldenPath/hg19/database/snp147.txt.gz resulting in pairs of personalised "maternal" and "paternal" chromosome files. To generate control data for a given chromosome, two 15x datasets were generated with ART using the corresponding paternal and maternal chromosome files as templates and combined to give a single 30x control dataset. To generate tumour data for a given chromosome, the corresponding paternal and maternal chromosome files were copied, and 250 sSRSCs were added to copied file. A minimum distance of at least 500 bp between each added sSRSC was enforced to avoid merging and coordinates that resulted sSRSC entering centromeric regions were avoided (centromeric regions defined in http://hgdownload.cse.ucsc.edu/goldenPath/hg19/database/cytoBand.txt.gz). Apart from these constraints, each added sSRSC's coordinate was determined randomly. Furthermore, for each added sSRSC its size, k, was randomly chosen from a uniform distribution, where $1 \leq k \leq 20$. For a given chromosome these sSRSC-containing paternal and maternal chromosome files were used as templates to generate two 15x using ART and were then combined into a single 30x tumour dataset.

1.3 Method 3: Removing false positives arising from somatic indels

We developed *indel filter*, to remove false positives caused by somatic indels. When tumour-consensus sequence pairs are examined during the SNV calling stage of GeDi the sequences within a pair must be aligned to one another. Accordingly, during this stage, pairs covering somatic indels will be misaligned unless a correcting gap is added across the indel site. If no correcting gap is added, sometimes this misalignment can giv e the appearance of multiple single character mismatches which GeDi would incorrectly call as SNVs. Indel filter removes false positives by identifying and discarding consensus sequence pairs covering somatic indels; these are often extracted during

suffix array-based SNV detection. Indel filter identifies pairs covering somatic indels by performing gapped pair-wise alignment of tumour-consensus sequence pairs using smith-waterman. A scoring regime of match score, mismatch penalty, gap open penalty and gap extension penalty set to 1, 1, 0, 0 respectively minimises gap insertion/extension penalties. Under this scoring regime, consensus pairs producing single-gapped pair-wise alignments are discarded, as such alignments are indicative of somatic indels. Supplementary Data, Table 1 shows the effect of indel filter on false positive reduction in the presence of somatic indels. In future work GeDi will call somatic indels, rather than discarding them.

1.4 Method 4: Benchmarking resource requirements

For all analyses each SNV caller was run on a machine with SGI UV2 scheduler and Xeon E5-4650v2 CPUs. GeDi was compiled with g++ version 4.8.5 with O3 optimisation. SMuFin was compiled with using the Makefile given in their release version, which uses gcc 4.8.5 (http://cg.bsc.es/smufin/). MuTect was run with Java(TM) SE Runtime Environment (version 1.6.0_45). The SGI UV2 scheduler allows process-specific user-definable resource allocation that is exclusively reserved for each process. Accordingly, for the results in rows 1-4 of Table 3 from the main paper: all SNV callers were allocated 200 GB of memory; GeDi and MuTect allocated 32 logical threads, and were run with their threading values set to 32 (Supplementary Data, Commands 3 and 4 provide example commands); the memory allocation pool of each Java environment running MuTect was set to 200GB (-Xmx200g); since SMuFin's parallelism is achieved through MPI, the 256 thread run of SMuFin (command given on SMuFin website http://cg.bsc.es/smufin) was allocated 16 compute nodes each with 16 threads whilst the 32 thread run of SMuFin was allocated 2 nodes with 16 threads each (Supplementary Data, Commands 1 and 2 in Section 4 show the exact commands use to run SMuFin in 256 and 32 thread mode respectively). For row 5 of Table 3 from the main paper (analysis of dataset MB:L.A), GeDi and MuTect were allocated 1500 GB of memory with 64 logical threads. Their commands were adjusted accordingly.

1.5 Method 5: Preprocessing and MuTect analysis of datasets TSD:chr17 and TSD:chr22

Preprocessing: Raw reads of TSD:chr17 and TSD:chr22 were quality checked using FASTQC v.0.11.5 and trimmed with Trimmomatic (Bolger *et al.*, 2014). These processed reads were then analysed by either MuTect or GeDi.

MuTect analysis: Trimmed reads were aligned to the reference genome (hg19 assembly) with BWA mem v.0.7.17 Li, Heng (2013) software with -v 1 -t 12 parameters. Aligned reads were converted to bam file then sorted and indexed using samtools v1.9. BAMs header were adjusted and processed with addOrReplaceReadGroups tool available in Picard suite v2.2.1 (http://broadinstitute.github.io/picard/) with parameters GID=1 RGLB=lib1 RGPL=illumina RGPU=unit1 RGSM=1. All duplicates in the files were removed using MarkDuplicates available in Picard suite v2.2.1. Data processing for mutation calling followed the GATK best Practises v.01-09-2018 which includes Indel Somatic mutation calling was performed using Mutect (Cibulskis *et al.*, 2013) with default parameters. SNPs annotated in 1000G_omni2.5.hg38.vcf were removed from the analysis and only the mutations tagged with "PASS" were retained.

1.6 Method 6: MB:L.A preprocessing

In Tyler S. Alioto (2015), preprocessing of MB:L.A raw sequencing reads followed best practices of the research group testing each pipeline. Information on these best practices is provided in the supplementary data of the work (Tyler S. Alioto, 2015). For pipelines MB.A, MB.B, MB.C, MB.D, MB.G, MB.H, MB.I, MB.J, MB.K, MB.L1, MB.L2, MB.M, MB.N, MB.O, MB.P, and MB.Q no details were specified on how the raw sequencing reads were processed. For pipelines MB.E, MB.F1, and MB.F2 the quality of MB:L.A raw sequencing reads was assesed using FastQC. This assessment lead to the trimming of the second reads of each 251bp MiSeq paired end run (runs A3MCW, A4DBD and A4DC6) being trimmed to 220bp (Tyler S. Alioto, 2015). Since the preprocessing practices of MB:L.A was only described for these pipelines, we decided to follow these practices exactly for our analysis of GeDi such that a fair comparison could be made between GeDi's and the aforementioned pipelines' output. Accordingly, we trimmed the reads of files:

- `_EGAR00001386984_ICGC_BM2_fastq_A3MCW_513F-B_GAGTGG_L001_R2_001.fastq.gz`
- `_EGAR00001386985_ICGC_BM2_fastq_A4DBD_513F-B_GAGTGG_L001_R2_001.fastq.gz`
- `_EGAR00001386986_ICGC_BM2_fastq_A4DC6_513F-B_GAGTGG_L001_R2_001.fastq.gz`
- `_EGAR00001386974_ICGC_BM2_fastq_A3MCW_514F-B_ATTCCT_L001_R2_001.fastq.gz`

- _EGAR00001386975_ICGC_BM2_fastq_A4DBD_514F-B_ATTCCT_L001_R2_001.fastq.gz
- _EGAR00001386976_ICGC_BM2_fastq_A4DC6_514F-B_ATTCCT_L001_R2_001.fastq.gz

from 251bp to 220bp using Trimmomatic (Bolger *et al.*, 2014) and left the rest of the data unchanged. Our own assessment of the raw sequencing data with FastQC agreed with the practices of MB.E, MB.F1 and MB.F2 - the second reads from runs A3MCW, A4DBD and A4DC6 should cleaved to 220bp, and the remaining data should be left unchanged.

1.7 Method 7: Calculation of percentage of GeDi calls within cis-regulatory or transcribed regions

SNV from GeDi ran in default mode when analysing MB:L.A were grouped into three catagories All_GeDi, sSRSC-residing SNV calls, and non_Gold_Set as described in the main text. A fourth group, Gold_Set, consists of all the SNVs from the Gold Set identified in Tyler S. Alioto (2015). SNVs in each of these groups were matched against a functional dataset consisting of transcribed and cis-regulatory regions. The transcribed regions included in the dataset comprise all introns and exons annotated in Human Reference Genome hg19. The cis-regulatory regions are the totality of DHS loci available from ENCODE Honey Badger DHS (https://personal.broadinstitute.org/meuleman/reg2map/).

Each of the four groups of SNVs was intersected with the functional dataset using bedtools intersect with default parameters to identify calls occuring in functional sites. The percentage of the SNV from each group that fell within the intersection was calculated and the results plotted in Figure 6 of main text.

2 Figures

2.1 Figure 1. Graphical explanation of GeDi's dual suffix array design.

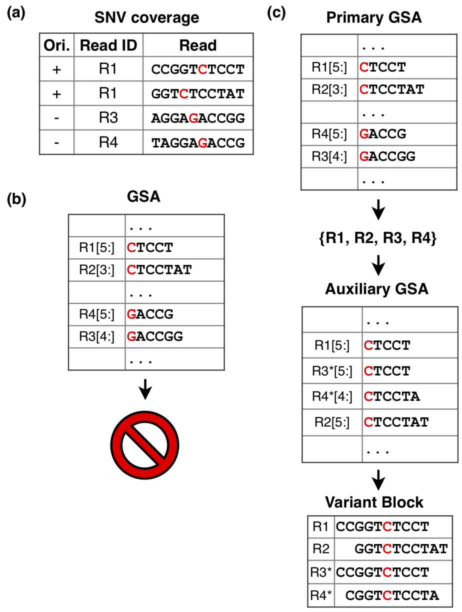

Figure 1: GeDi's dual suffix array design enables SNV detection at low allele frequency. a) A single SNV is covered by four reads R1-4, however only two reads cover the SNV in each DNA orientation (Denoted by +/- in Ori. column). b) In SMuFin's approach to suffix array-based SNV detection, a single suffix array is used (Moncunill *et al.*, 2014). In this suffix array, reads R1-2 form a separate tumour-suffix enriched section to R3-4. As neither of these sections contain at least $MSS = 4$ tumour-derived suffixes, reads R1-4 are not extracted, even though the SNV is genuine and has a total coverage $\geq MSS$. Hence, the SNV is undetectable. Since this SNVs coverage is low, its allele frequency is likely to be low. Therefore, the original GSA approach shows reduced sensitivity for SNV detection at low allele frequency. c) In GeDi's dual suffix array design, reads R1-4 are extracted from the primary GSA as $pMSS = 2$ and the separate tumour-suffix enriched sections formed from R1-2 and R3-4 both have $\geq pMMS$ tumour-suffixes. Once extracted, the auxiliary GSA groups together R1-4 with their reverse complements (Rn* denotes reverse complement of read Rn). Since the reverse complement grouping leads to tumour-suffix enriched sections with size $\geq aMSS = 4$, reads R1-4 are successfully extracted and the SNV is now detectable. The extracted reads R1-4 are now locally aligned into a variant block using the start positions of the suffixes within the enriched section. For a given read Rn, Rn[i:] is the suffix starting at position i in Rn.

2.2 Figure 2. Graphical explanation of GeDi's masking filter.

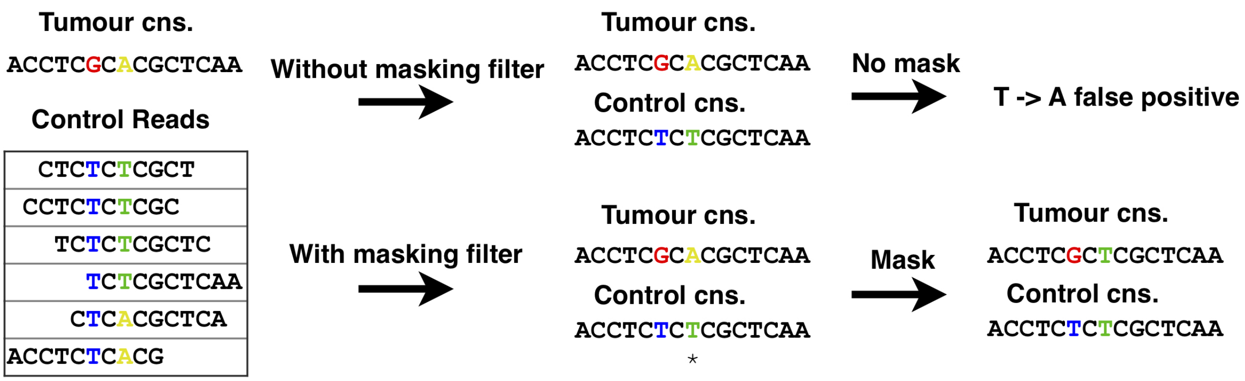

Figure 2: GeDi's masking filter reduces false positive calls caused by SNPs. Leftmost panel: The tumour consensus sequence (Tumour cns.) and the aligned control reads that will be used to make the control consensus sequence (Control cns.) are shown. An SNV is present at the coloured site (red 'G', blue 'T'). However, an SNP is also located in close proximity (yellow 'A', green 'T'). In the tumour consensus sequence, the 'A' SNP allele occurs on the same chromosome as the SNV. However in the control dataset, the 'T' allele is most frequent. Middle panel: Without the masking filter, the 'T' allele (most frequent) was the chosen allele for the control consensus sequence. With the masking filter, although the 'T' allele is selected, the masking filter identified the position for masking (asterisk), due to their being a high frequency of two bases at the SNP position (2 A's and 4 T's) and hence, greater than one non-zero component within the SNP containing column of the control sequence's frequency matrix. Rightmost panel: Without masking, the T/A SNP would be reported as an SNV by GeDi resulting in a false positive call. With masking, the 'A' within the tumour consensus sequence is replaced with the 'T' in the control. As a result, a false positive call is avoided. Since the tumour consensus sequence is never aligned, masking does not influence the coordinate position of the called SNV.

2.3 Figure 3-6. IGV snapshots of SNV calls made by GeDi when analyzing dataset TSD:chr22.

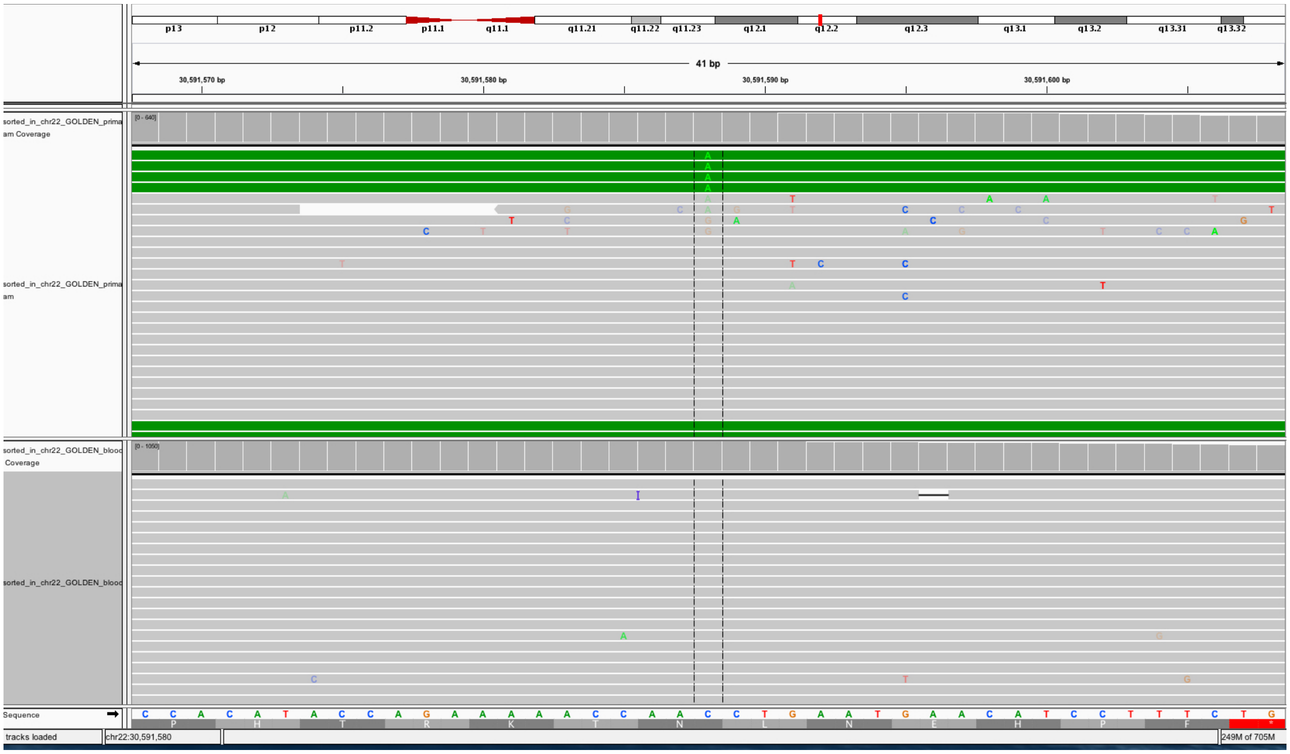

Figure 3: IGV Snapshot of aligned reads from TSD:chr22 dataset at hg19 genome coordinate chr22:30591588 (http://www.broadinstitute.org/igv).

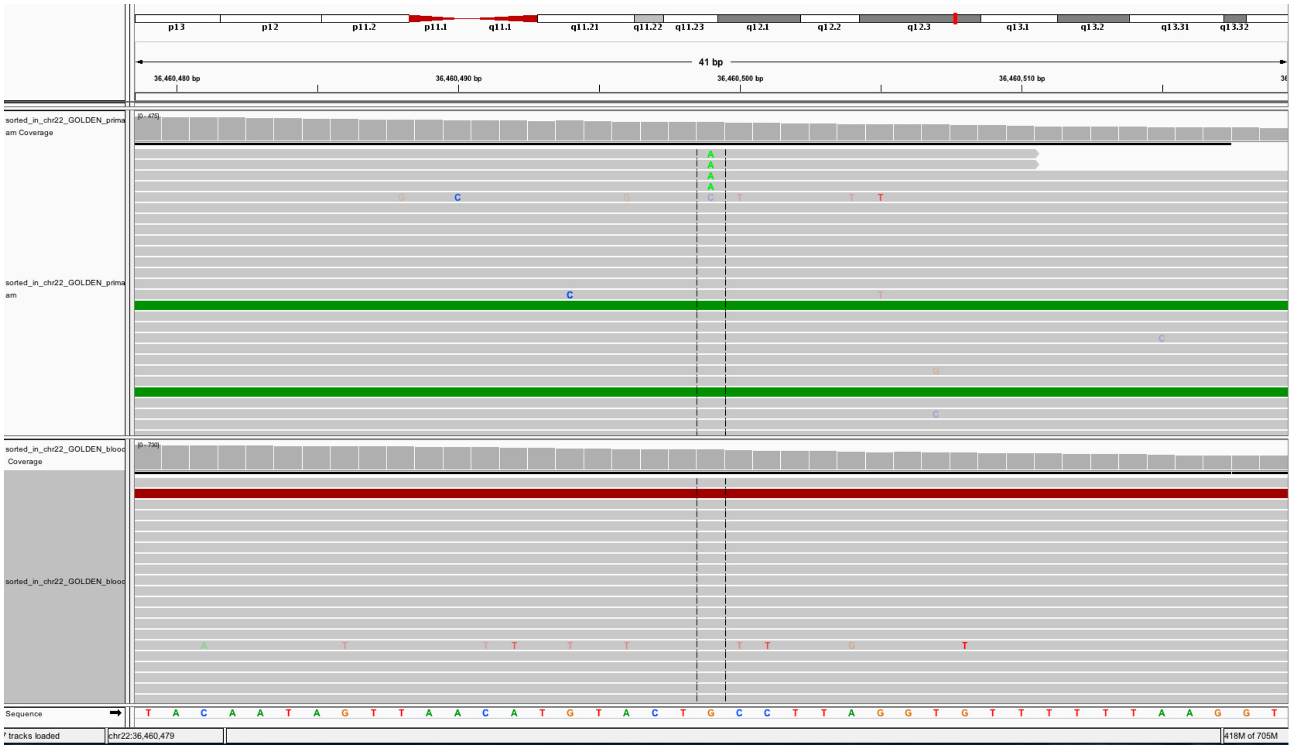

Figure 4: IGV Snapshot of aligned reads from TSD:chr22 dataset at hg19 genome coordinate chr22:36460499.

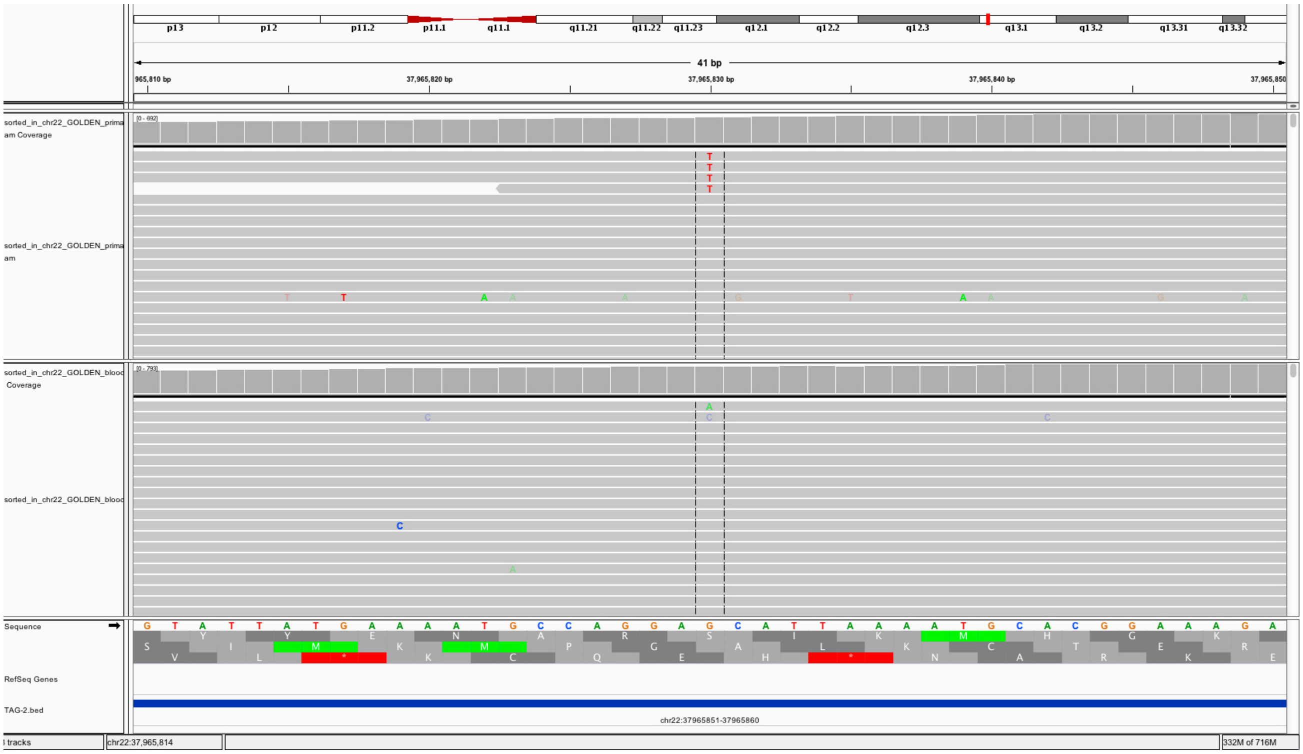

Figure 5: IGV Snapshot of aligned reads from TSD:chr22 dataset at hg19 genome coordinate chr22:37965830.

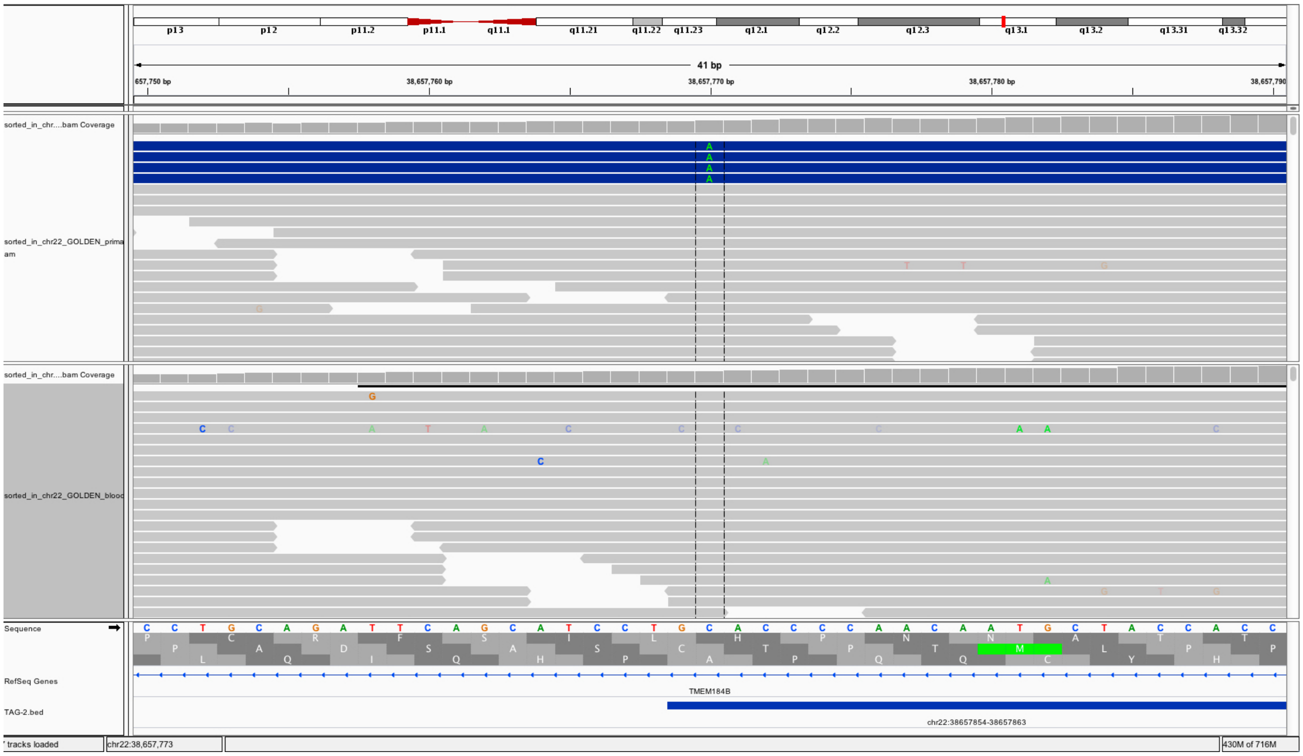

Figure 6: IGV Snapshot of aligned reads from TSD:chr22 dataset at hg19 genome coordinate chr22:38657770.

2.4 Figure 7. Effect of emfilter on recall and precision for SNV calling across different sSRSC.

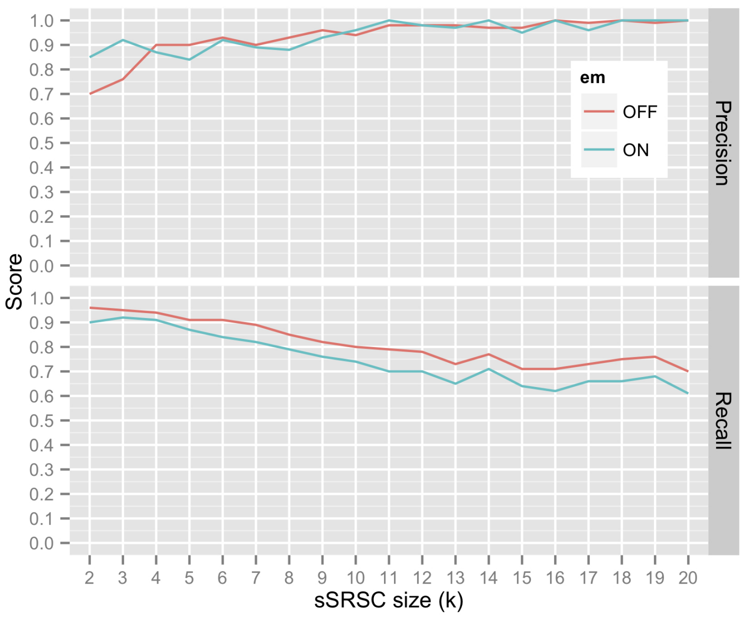

Figure 7: We analyzed the same datasets used to determine GeDi's an d MuTect's precision and recall for sSRSC detection (Figure 4 of main paper) with GeDi in default mode, with and without emfilter; a full explanation of how these datasets were generated is given in Supplementary Data, Method 2. The plot shows the *k-precision* and *k-recall* attained by GeDi for each sSRSC of size $2 \geq k \geq 20$ with emfilter on (ON:blue) and off (OFF:red). We can see that, although emfilter reduces sensitivity, unlike with reference-based SNV callers, the loss in sensitivity is not proportional to the size of the sSRSC (Figure 4 of main paper). Hence, emfilter does not cause the mapping-associated sensitivity loss that afflicts reference-based SNV callers.

3 Tables

3.1 Effect of indel filter on false positive reduction.

Table 1: Effect of indel filter. Two 30x simulated tumour-control paired NGS datasets were generated with ART. One dataset (dataset No indels in table) was built using hg19 chromosome 22 (extracted from from ftp://ftp.broadinstitute.org/bundle/hg19/ucsc.hg19.fasta.gz) as an input template to ART and contained no somatic indels. The other dataset (dataset Indels in table) was built by combining two 15x sub-datasets, each generated by ART; one sub-dataset used hg19 chromosome 22 (downloaded from the aforementioned link) as the input template, the other used a copy of hg19 chromosome 22, identical to the original apart from the presence of 100 randomly located indels with a maximum character length of 10. Hence, the Indels dataset contains somatic indels with an average allele frequency of 0.5. No SNVs were added to either dataset. These datasets were analysed with GeDi in default mode, with and without the indel filter (Treatment: ON and OFF respectively). The number of false positives (FP) for all runs was recorded and are given in the table below. We can see that in the presence of indels, indel filter reduces the number of false positives.

Dataset	Treatment	FP
No indels	ON	0
Indels	ON	4
No indels	OFF	4
Indels	OFF	18

3.2 Effect of masking filter on false positive reduction.

Table 2: Effect of masking and multi-locus filter. A 30x simulated tumour-control paired NGS dataset was generated with ART. The control dataset was built using hg19 chromosome 22 (extracted from ftp://ftp.broadinstitute.org/bundle/hg19/ucsc.hg19.fasta.gz) as an input template into ART. The tumour dataset was built from two 15x sub-datasets, each generated by ART; one sub-dataset used hg19 chromosome 22 extracted from the aforementioned file as an input template into ART, the other using a copy of hg19 chromosome 22 with sequence identical to the original apart from the presence of 200 randomly located SNVs as an input template into ART. We analysed this paired tumour-control dataset with GeDi, with both filters on (Mask ON, Multi-locus ON), mask filter off (Mask OFF, Multi-locus ON), multi-locus off (MASK ON, Multi-locus OFF), and both off (MASK OFF, Multi-locus OFF). The number of false positives (FP) for all runs was recorded and is given in this table.

Treatment	FP
Mask ON, Multi-locus ON	15
Mask OFF, Multi-locus ON	165
Mask ON, Multi-locus OFF	351
Mask OFF, Multi-locus OFF	1311

3.3 Call information of SNV calls made by GeDi when analyzing dataset TSD:chr22.

Table 3: Coverage and allele frequency of the four SNV calls made by GeDi and not made by MuTect for dataset TSD:chr22. The genome coordinate of each mutation is given (hg19 coordinate) along with the control and tumour variant called by GeDi. Total coverage is the total number of reads covering a given coordinate. Variant coverage is the number of reads with the variant allele covering a given coordinate. Variant allele frequency is the ratio of variant coverage to total coverage.

Genome Coordinate (hg19)	Control Variant	Tumour Variant	Total Coverage (tumour / control)	Variant Coverage (tumour / control)	Variant Allele Frequency (tumour)
chr22:30591588	C	A	623 / 1026	7 / 1	0.011 (1.1%)
chr22:36460499	G	A	285 / 477	6 / 0	0.021 (2.1%)
chr22:37965830	G	T	573 / 690	5 / 1	0.009 (0.9%)
chr22:38657770	C	A	103 / 149	4 / 0	0.039 (3.9%)

3.4 SNV calls made by GeDi when combining output from runs with pMSS = 1 and pMSS = 4.

Table 4: Number of GeDi and MuTect SNV calls for datasets TSD:chr22 and TSD:chr17 when combining calls from GeDi run with $pMSS = 1$ and $pMSS = 4$. We analysed this dataset twice with GeDi, setting $pMSS$ to 1 and 4 for the different runs. We then computed the recall and precision on the union of these call files. The tabel given shows the number of calls made by MuTect and GeDi when taking the union of the two runs. The column titled Intersection describes the number of SNV calls MuTect and GeDi made in common.

Dataset	Mutect Calls	GeDi Calls	Intersection
TSD:chr22	9	13	9
TSD:chr17	40	76	37

4 Commands

Commands 1-4: Example commands used for GeDi, MuTect and SMuFin when running resource requirement analysis. Dataset downloaded from http://cg.bsc.es/smufin/ is used as inputs. For commands 5 and 6, all parameters in angled brackets would be set appropriately, the remaining parameters were never changed.

1.
```
mpirun --np 16 ./SMuFin --ref ref_genome/hg19.fa \
    --normal_fastq_1 normal_fastqs_1.txt --normal_fastq_2 normal_fastqs_2.txt \
    --tumor_fastq_1 tumor_fastqs_1.txt --tumor_fastq_2 tumor_fastqs_2.txt \
    --patient_id chr22_insilico --cpus_per_node 16
```

2.
```
mpirun --np 2 ./SMuFin --ref ref_genome/hg19.fa \
    --normal_fastq_1 normal_fastqs_1.txt --normal_fastq_2 normal_fastqs_2.txt \
    --tumor_fastq_1 tumor_fastqs_1.txt --tumor_fastq_2 tumor_fastqs_2.txt \
    --patient_id chr22_insilico --cpus_per_node 16
```

3.
```
./GeDi -c chr22 -v 30 -t 32 -i ./smufin_dataset_fastqs.txt \
    -x ./bowtie_indexes/ucsc.hg19.fasta  -o gedi_analysis
```

4.
```
./jdk/jdk1.6.0_45/bin/java -Xmx200g -jar \
    /work/ic711/mutect/muTect-1.1.4.jar --analysis_type MuTect \
    --num_threads 32 --reference_sequence ./bowtie_indexes/ucsc.hg19.fasta \
    --input_file:normal smufin_control.bam --input_file:tumor smufin_tumour.bam \
    --out mutect_smufin_dataset_out
```

5.
```
bowtie2 -p 16 -x \
    ./bowtie_indexes/ucsc.hg19.fasta -U <data.fastq> -S <data.sam>
```

6.
```
./art_illumina -i <template.fasta> -mp -m 2500 -s 0 \
    -rs $seed -l 100 -ss HS25 -f <coverage> -o <output_base_name>
```

References

Bolger, A. M., Lohse, M., and Usadel, B. (2014). Trimmomatic: a flexible trimmer for Illumina sequence data. *BIOINFORMATICS*, **30**(15), 2114–2120.

Cibulskis, K., Lawrence, M. S., Carter, S. L., Sivachenko, A., Jaffe, D., Sougnez, C., Gabriel, S., Meyerson, M., Lander, E. S., and Getz, G. (2013). Sensitive detection of somatic point mutations in impure and heterogeneous cancer samples. *Nature biotechnology*, **31**(3), 213–219. PT: J; UT: WOS:000316439500014.

Huang, W., Li, L., Myers, J. R., and Marth, G. T. (2012). Art: a next-generation sequencing read simulator. *Bioinformatics*, **28**(4), 593–594. PT: J; UT: WOS:000300490500023.

Li, Heng (2013). Aligning sequence reads, clone sequences and assembly contigs with BWA-MEM. *arXiv*.

Moncunill, V., Gonzalez, S., Bea, S., Andrieux, L. O., Salaverria, I., Royo, C., Martinez, L., Puiggros, M., Segura-Wang, M., Stuetz, A. M., Navarro, A., Royo, R., Gelpi, J. L., Gut, I. G., Lopez-Otin, C., Orozco, M., Korbel, J., Campo, E., Puente, X. S., and Torrents, D. (2014). Comprehensive characterization of complex structural variations in cancer by directly comparing genome sequence reads. *Nature biotechnology*, **32**(11), 1106–1112. PT: J; TC: 9; UT: WOS:000344977000015.

Tyler S. Alioto, e. a. (2015). A comprehensive assessment of somatic mutation detection in cancer using whole-genome sequencing. *Nature Communications*, **6**, 10001. PT: J; UT: WOS:000367579200001.
